# The clinical utility of imaging in osteoarthritis and its importance in future prediction of total hip replacement; a nested case-control study within the AGES-Reykjavik cohort

**DOI:** 10.1093/jbmr/zjaf202

**Published:** 2025-12-27

**Authors:** Kenneth E S Poole, Ilya S Burkov, Graham M Treece, Andrew H Gee, Fjola Johannesdottir, Simona D’Amore, Stephen K Kaptoge, Sigurdur Sigurdsson, Thor Aspelund, Tamara B Harris, Helgi Jonsson, Vilmundur Gudnason, Thomas D Turmezei

**Affiliations:** Department of Medicine, University of Cambridge, Addenbrooke's Hospital, Cambridge CB2 0QQ, United Kingdom; Department of Medicine, University of Cambridge, Addenbrooke's Hospital, Cambridge CB2 0QQ, United Kingdom; Department of Engineering, University of Cambridge, Cambridge CB2 1TN, United Kingdom; Department of Public Health and Primary Care, University of Cambridge, Cambridge CB1 8RN, United Kingdom; Department of Medicine, University of Cambridge, Addenbrooke's Hospital, Cambridge CB2 0QQ, United Kingdom; Department of Medicine, University of Cambridge, Addenbrooke's Hospital, Cambridge CB2 0QQ, United Kingdom; Department of Public Health and Primary Care, University of Cambridge, Cambridge CB1 8RN, United Kingdom; The Icelandic Heart Association, Kópavogur, Iceland, 201; The Icelandic Heart Association, Kópavogur, Iceland, 201; Public Health Sciences, University of Iceland, Reykjavík, Iceland, 101; Laboratory of Epidemiology and Population Sciences, Intramural Research Program, National Institute on Aging, Baltimore 21224, United States; Public Health Sciences, University of Iceland, Reykjavík, Iceland, 101; Landspitali University Hospital, Reykjavík, Iceland, 101; The Icelandic Heart Association, Kópavogur, Iceland, 201; Faculty of Medicine, University of Iceland, Reykjavík, Iceland, 101; Department of Medicine, University of Cambridge, Cambridge CB2 0QQ, United Kingdom; Department of Radiology, Norfolk and Norwich University Hospital, Norwich NR4 7UY, United Kingdom

**Keywords:** Osteoarthritis, Total Hip Replacement, Imaging, Computed Tomography, Implants

## Abstract

Current guidelines are split on the role that imaging has in the clinical assessment of osteoarthritis (OA), yet clinical CT imaging has now revealed how a 3D approach can improve prediction of TH replacement (THR) over 2D measures alone. We applied 2D grading and measurement along with 3D cortical bone mapping to ordinary clinical CT imaging of the pelvis in a cohort of healthy older people, aiming to discover which of these features had clinical utility in predicting THR within 8 yr and which were related to baseline hip pain. Using a nested case-control design in the Age, Gene/Environment Susceptibility-Reykjavik (AGES-Reykjavik) study, 74 future THR cases were age and sex-matched with 184 controls from the cohort (age 74 ± 5 yr). Baseline assessment involved a validated hip pain questionnaire and pelvic CT. The following were performance-tested using receiver operating characteristics analysis and clinical utility index: (1) hip pain, (2) Kellgren and Lawrence grade (K&L grade), (3) minimum joint space width, and (4) 3D cortical bone thickness (CTh). The clinical utility index for prediction of future THR from baseline pain was poor at 0.28, with the inclusion of imaging improving this to 0.79 (K&L grade) and 0.82 (3D CTh). Self-reported hip pain at baseline was also a poor-to-marginal predictor of THR (AUC = 0.63), but 3D cortical thickening at the femoral head was predictive of future THR (0.81). Having radiographic OA strongly predicted THR irrespective of hip pain (0.85). Combining hip pain, K&L grade, and 3D CTh gave optimal prediction (0.88). Ascertainment bias may have occurred, if primary care physicians requested their own radiographs of their patients’ hips. Imaging features from standard clinical CT identifies patients at high risk of progression to surgery for OA, regardless of baseline pain.

## Introduction

Hip osteoarthritis (OA) is a highly prevalent and disabling joint disease with a significant individual and societal impact.^1^^,^^2^ In the USA, there has been a 20% increase in reported cases of OA in the decade since 2002,^3^ and from 2005 to 2030 total hip replacements (THRs) for OA are projected to increase by 174%.^4^ Patients destined for THR typically have groin or medial thigh pain, stiffness, reduced hip movement on examination, but also a narrowed radiographic joint space.^5–7^ Although the decision to offer THR depends partly upon patient-reported measures, such as pain and functional limitation, the quality of secondary care referral of patients with hip OA might be enhanced if researchers could define specific and sensitive OA imaging features that have clinical utility.^8^^,^^9^

Much is already known about the utility of imaging in the clinical assessment of OA. Seven years before THR, the baseline grade of radiographic hip OA was the strongest predictor of clinical progression, with the odds ratio (OR) for THR increasing from 6 (irrespective of baseline pain) to 24 (95% CI, 11-52) in those having both pain and radiographic OA.^10^ In Iceland, where we have focused our studies, the hazard ratio of radiographic hip OA for incident THR over 11-28 yr was 12.9 (95% CI, 7.9-21), regardless of symptoms.^11^ Lievense et al. found that the radiographic structural features of OA such as narrowing of the hip joint space were the key predictors of eventual THR (alongside age >60 yr, pain and restricted hip movement) in a large sample of adults presenting to primary care with hip pain and followed for 3-6 yr.^7^ Predicting individuals at risk of progression to future surgical intervention is also an important step in identifying patients that might stand to benefit from currently available non-surgical therapies such as stem cell treatments.

Recommendations concerning how and when to request radiographs in diagnosis and referral vary across the major evidence-based guidelines.^6^^,^^12–14^ Some guidelines recommend an AP hip radiograph before, and to help guide surgical referral,^12^^,^^15^ while others recommend imaging for patients with atypical presentations and unexpected rapid progression to identify alternative diagnoses.^14^ Conversely, the authors of the UK NICE Osteoarthritis Quality Standard 87 (linked to guideline CG177) describe radiographs as *unnecessary*, *potentially harmful*, and *costly*, mandating that doctors do not request *any* imaging for hip OA diagnosis, management, or when considering referral.^16^ Despite this, there remains discordance between guideline and practice; many clinicians (cognizant perhaps, of *spectrum bias*^13^) do find hip radiographs to be useful when deciding about referral and treatment.

Researchers have shown that radiological hip OA is characterized by highly focal bony changes on the surface of the femoral head,^17^ and proposed that increased cortical thickness (CTh) could predate the development of painful disabling hip OA.^18^ Recently, a narrow 3D joint space (JSW) mapped from CT images showed optimal prediction of future THR in a cohort of healthy older adults when combined with 3D shape information and radiographic Kellgren and Lawrence (K&L) grading.^19^ In the ongoing debate over the role of imaging in the care of patients with OA, these studies suggest that CT has a facilitatory role in the clinical assessment of hip OA.

In the present study, we aimed to discover which imaging features of hip OA (if any) had clinical utility in determining future THR for hip pain. Using a large, prospective, nested case-control study design within a healthy random sample of Icelandic men and women, we investigated 2D imaging measures, such as minimal joint space width (mJSW) and K&L grade along with 3D cortical bone thickness (CTh) from standard clinical CT imaging.

## Materials and methods

### Study population

**Figure 1:**
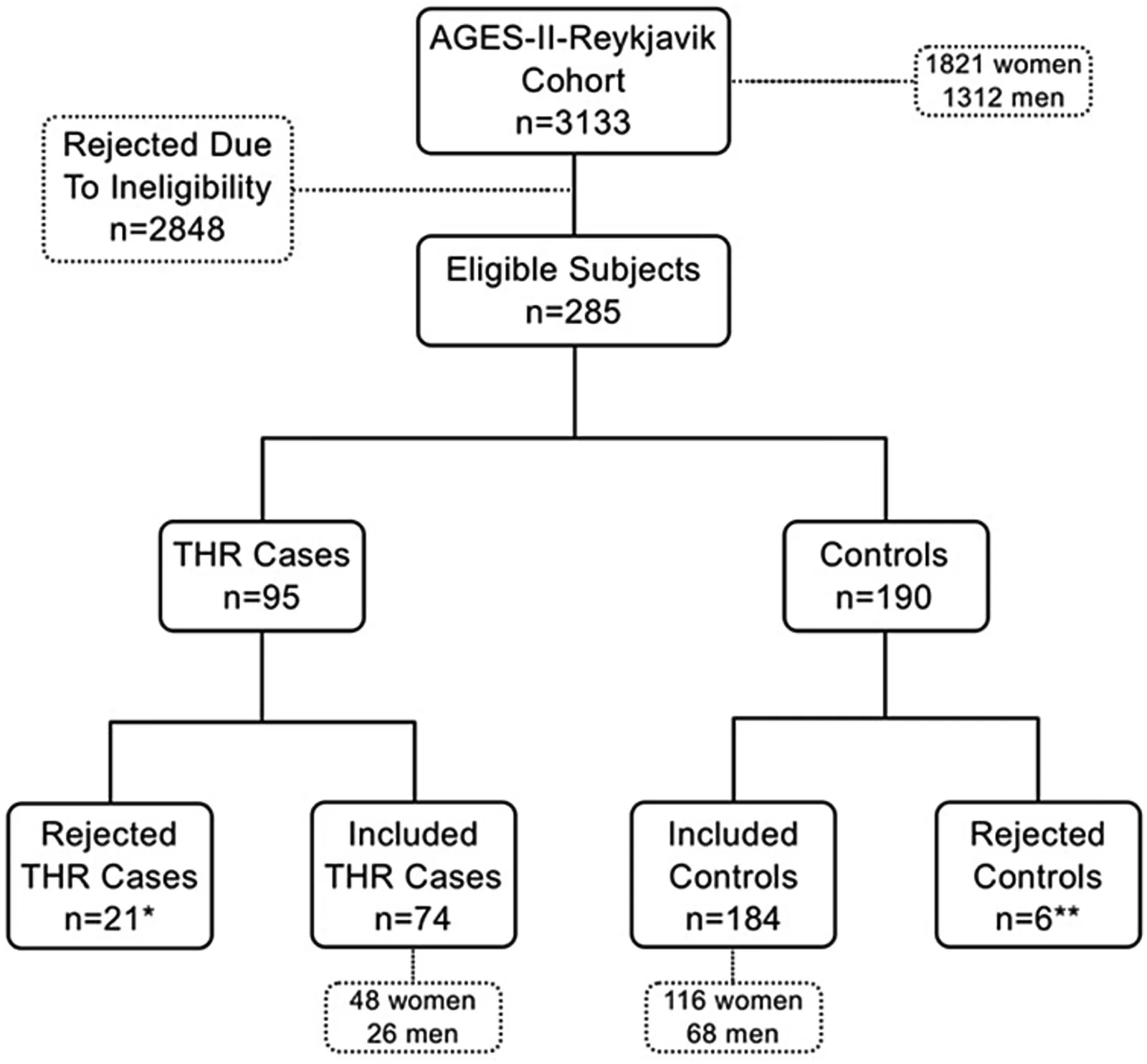
Study flowchart showing the nested case-control design within the large healthy prospective aging cohort. * 21 rejected: 18 THR not due to OA, 2x incomplete scans, and 1x motion artifacts. ** 6 rejected: 4x motion artifact, 1x incomplete scan, and 1x unreliable phantom density value.

Participants (*n* = 3133) were volunteers from Age, Gene/Environment Susceptibility-Reykjavik (AGES-Reykjavik), a single-center prospective population study, which has been well described.^20^ AGES participants were invited to attend the Icelandic Heart Association (Hjartavernd) for extensive phenotyping, including CT imaging. Study subjects were drawn from the 5764 (58% female) survivors of the original sample of the population of Reykjavik born in 1907-1935, on whom studies began in 1967. Discharge records from all hospitals performing THR in Iceland were examined. Cases were reviewed by a rheumatologist (H.J.) who confirmed that THR was for OA only. The nested case-control flowchart is shown in Figure 1. A similar selection of participants was also used in the 2020 study by Turmezei et al. looking at the role of 3D JSW in prediction of future THR.^19^

Baseline assessment of all 3133 volunteers included pelvic CT (Somaton Sensation 4, Siemens Medical Systems), with imaging of both hips from 1 cm above acetabular margin to >3 mm below the lesser trochanter acquired in the supine position. Spiral acquisition parameters were: 120 kVp, 140 mAs, and pitch = 1. Axial frames were reconstructed with 1 mm slice thickness, 512 × 512 matrix in a 50 cm field of view, and x-y pixel dimensions of 0.977 mm with a standard soft tissue kernel. Hip-pain specific questions were from the Western Ontario and McMaster Universities WOMAC and included “HEALHIP12. In the past 12 months, have you had pain in or around either hip joint, including the buttock, groin, or either side of the upper thigh, lasting at least 1 mo?” During a maximum of 8 yr (average 5 yr) following participants who underwent baseline CT, 95 THR were reported. After screening, 74 subjects remained (48 F and 26 M), who had undergone 90 THR operations (16 bilateral THRs). Using a random selection procedure, at least 2 controls were selected for each THR case matched for year of recruitment, sex and age. After screening, 184 subjects remained (116 women and 68 men), giving a total of 258 included participants (Figure 1). K&L grade assessment, mJSW measurement, and 3D OA disease feature grading were performed on digitally reconstructed radiographs (DRRs) (Table 1).^17^^,^^21^ DRR creation and cortical bone mapping methods are shown in table 1.^22–24^ The single operator (TDT) performing the mJSW scores previously showed substantial intra-observer reliability (0.63, 95% CI, 0.37-0.90) weighted kappa statistic for this methodology.^17^

**Table 1:** Imaging techniques and osteoarthritis grading.

**Digital reconstructed radiographs and K&L grading**	Single experienced MSK radiologist (TDT) created Digital Reconstructed Radiograph (DRRs) (13) TDT blinded to THR status/clinical outcomes and pain status PACS viewing software (OsiriX, Pixmeo Sarl; v.3.9.3). Multiplanar reformatting (MPR) of mean intensity projection slab at each hip with a soft tissue window Ensured coronal coverage of anterior and posterior joint margins Coronal depth thickness of up to 8 cm DRR simulated standard pelvic radiograph (example below) K&L grading and mJSW performed at 200% magnification 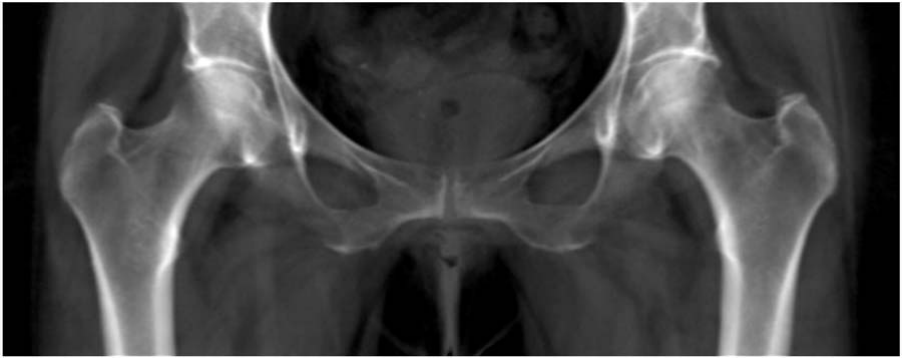
**3D cortical bone mapping (CBM)**	Cortical thickness measured across the proximal femur using cortical bone mapping (Stradwin software) Each proximal femur segmented into a surface from a triangular mesh of vertices **{1-3}** Thickness measured to an accuracy of approximately 0.3 mm (mean±SD error 0.01±0.58 mm) **{4}** Cortical surface measurement locations co-registered onto single canonical femur **{5}** Location and magnitude of patches of bone thickening associated with future THR highlighted by statistical parametric mapping, SPM**{6}** Detailed statistical methods regarding the 3D CBM technique and methods are in (18-20) General linear model (GLM) for SPM allowed for group (case/control), age, sex, weight, height, size (shape mode 0) and the first six non-rigid shape modes (SM), with Surfstat package in MATLAB GLM CTh = 1 + group + age + sex + weight + height + SM0 + SM1 + SM2 + SM3 + SM4 + SM5 Two-tailed T-testing across the cohort for uncorrected *p*-values at each measurement location Random field theory gave *p*-values corrected for multiple comparisons (controlling for type I errors) Average thickness calculated from the significant “patch” in each individual used in logistic regression
	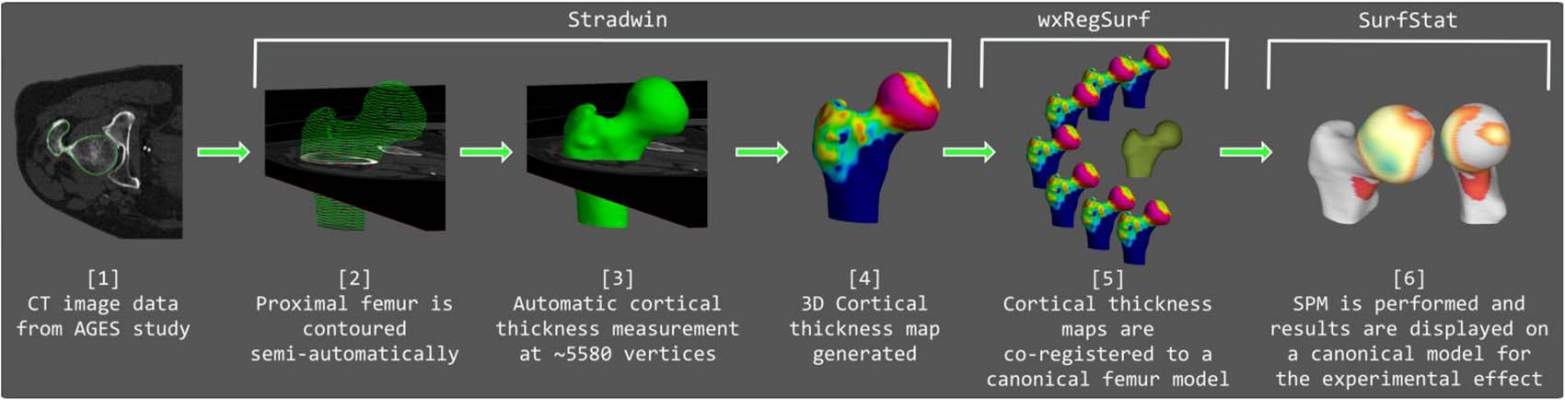

The study was approved (VSN 00-063) by the National Bioethics Committee in Iceland as well as the National Institute of Aging with all participants having provided written informed consent. The full protocol for this sub-study was approved by the Icelandic Heart Association steering group.

### Statistical methods

Statistical analyses were conducted using MATLAB R2016b (The MathsWork, Inc.), JMP (v13.0 SAS Institute Inc.), and Stata Statistical Software (v14.2 StataCorp). Baseline measures were hip pain (HEALHIP12, K&L Grade, mJSW, and mean 3D CTh) from the significant ROI patch from statistical parametric mapping (SPM) (see Table 1). Logistic regression determined the OR for THR expressed per each SD increase in CTh or decrease in mJSW and per increment in grade of K&L with adjustment for age, sex, height, baseline pain, femur shape, and using leave-one-out cross-validation. The area under the receiver operating characteristic (AUC, ROC) curve was used to assess the performance of the model. Receiver operating characteristic curves cut-offs were derived with JMP and the relationships between the various OA grades or increasing structural/imaging evidence of OA and hip pain were examined with nominal logistic fit methods. MATLAB was used to perform leave-one-out cross-validation of AUC values. Performance of the various logistic regression models, including imaging performance with and without hip pain was compared in STATA using the roccomp command and chi square analysis. Contingency analysis and positive and negative clinical utility index (UI) (*UI+* and *UI−*) was used to estimate the performance of diagnostic cut offs in regard to imaging utility in individuals presenting with baseline pain.^9^ We modeled THR as a binary outcome using logistic regression because our primary aim was to compare baseline discriminative performance (AUC, UI+/−) of imaging/clinical measures to inform clinical decision-making and to simulate how imaging could aid patient stratification at the time of assessment. Although follow-up was variable, cases and their matched controls were selected to be comparable on recruitment year, age and sex, which reduces systematic differences in follow-up distribution between groups. We acknowledge that formal Cox modeling would provide estimates of hazard over time, but THR was an uncommon outcome in the whole cohort, with only 74/3133 (2.4%) cases recorded over 8 yr of follow-up. Therefore, it would be likely that time-to-event analysis would give similar results as logistic regression.

## Results

Patients were well matched at baseline (Table 2). Two thirds of the participants destined for THR were female. The mean age of participants was 74.3 ± 4.7 yr and mean BMI was 27.7 ± 4.2 kg/m^2^. Cases underwent THR 35.8 mo (±23.7, range 1-96 mo) after their baseline CT*.* There were no significant differences in age, height, weight, and BMI between the control group and THR group, respectively (Table 2). Considering case hip DRRs, 65% of those graded had OA by K&L ≥2 at baseline vs 10% of control hip DRRs (Table 2).

**Table 2:** Baseline variables in study cases (who eventually underwent THR for osteoarthritis) and matched controls.

**Baseline data**	**Controls**	**Cases**	**Female controls**	**Female cases**	**Male** **controls**	**Male** **cases**
**Number of subjects (%)** ^**a**^	**184 (71%)**	**74 (29%)**	116	48	68	26
**Age ± SD (yr)**	**74.0 ± 4.7**	**74.0 ± 4.7**	74.1 ± 5.0	73.6 ± 4.6	74.8 ± 4.6	74.8 ± 4.9
**Height ± SD (m)**	**1.68 ± 0.09**	**1.67 ± 0.09**	1.62 ± 0.05	1.62 ± 0.06	1.77 ± 0.07	1.75 ± 0.06
**Weight ± SD (kg)**	**77.5 ± 13.6**	**78.1 ± 12.7**	73.1 ± 12.8	76.5 ± 12.0	85.0 ± 11.6	80.9 ± 13.6
**Cortical bone thickness in femoral patch** ^**(from CBM** **Figure 2**)^ **± SD (mm)**	**1.1 ± 0.1** ^b^	**1.3 ± 0.2** ^b^	1.1 (± 0.1)^b^	1.3 (± 0.2)^b^	1.1 (± 0.1)^b^	1.4 (± 0.2)^b^
**mJSW from DRR median** **+ IQR (mm)**	**2.3** (1.8, 2.8)^b^	**1.1** (0.6, 1.9)^b^	2.0 (1.5, 2.6)^b^	1.1 (0.4, 1.8)^b^	2.6 (2.0, 3.0)^b^	1.6 (1.0, 2.6)^b^
**Hip pain in the 12 mo prior to imaging investigation**	**19 (10%)**	**33 (45%)**	15 (13%)	24 (50%)	4 (6%)	9 (35%)
**Number of hips, (baseline DRR analyzed)**	**368**	**90**	232	59	136	31
**KLG 0; no OA**	**279 (76%)**	**18 (20%)**	170 (73%)	9 (15%)	109 (80%)	9 (29%)
**KLG 1; doubtful OA**	**53 (14%)**	**13 (15%)**	34 (15%)	8 (14%)	19 (14%)	5 (16%)
**KLG 2; mild OA**	**26 (7%)**	**19 (21%)**	20 (9%)	13 (22%)	6 (4%)	6 (19%)
**KLG 3; moderate OA**	**10 (3%)**	**30 (33%)**	8 (3%)	23 (39%)	2 (2%)	7 (23%)
**KLG 4; severe OA**	**0 (%)**	**10 (11%)**	0 (0%)	6 (10%)	0 (0%)	4 (13%)

a Entire dataset.

b *p <* .0001 case vs control.

Bold values indicate full cohort (both sexes).

### Cortical bone mapping

**Figure 2:**
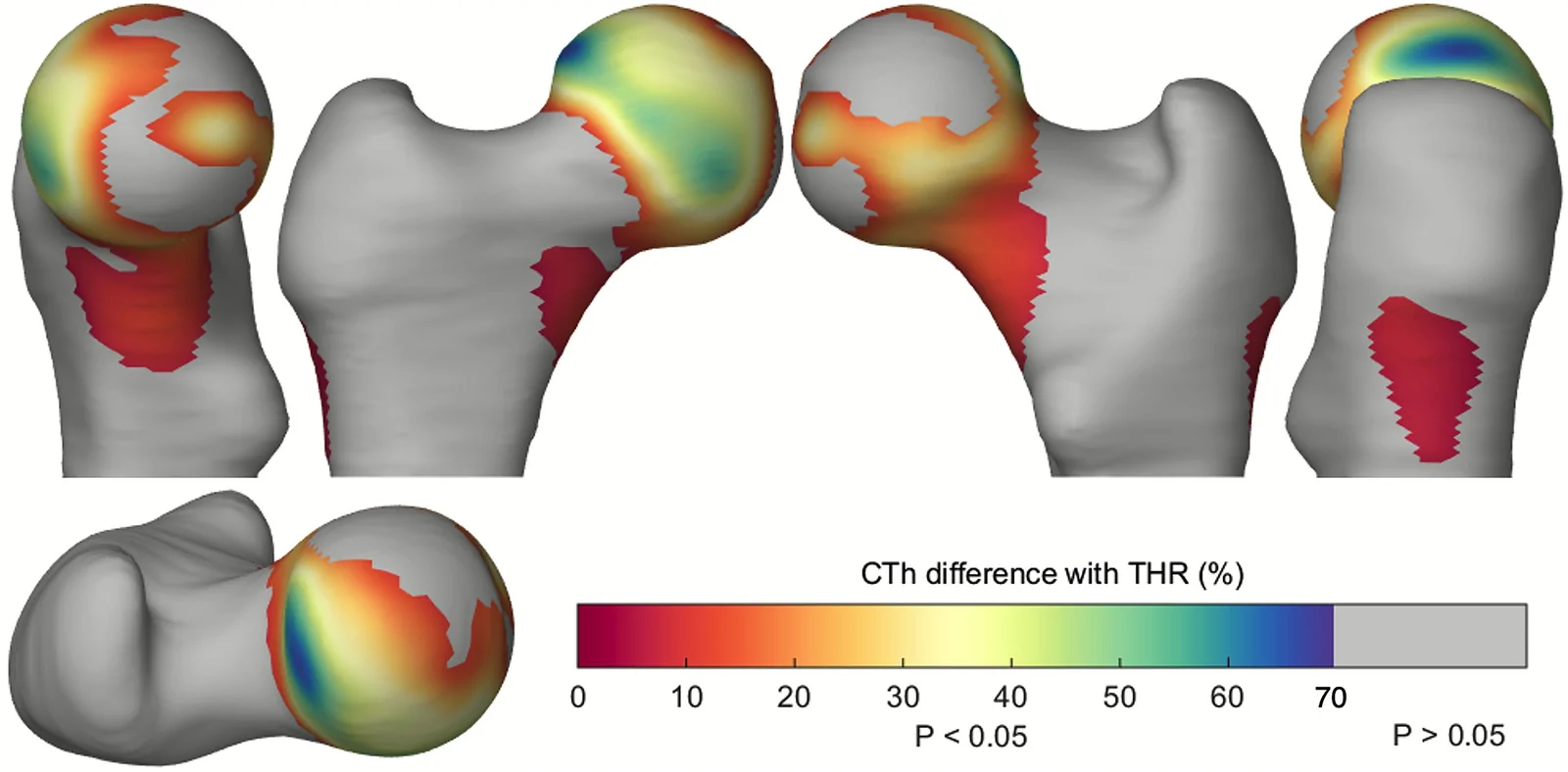
Statistical parametric mapping results. Percentage differences in baseline cortical thickness between subjects who went on to have THR and matched controls. Displayed as a statistical difference map with a color scale for the magnitude (%) of the relative differences. Gray areas are regions where there was no statistical significance between THR patients and controls. Blue, yellow, and orange areas demonstrate 35%-70% thicker femoral head bone in the focal areas. The average cortical thickness from within the multicolored ROI was used for further analysis, with values and SD shown in Table 2. The model containing only the average CTh from this ROI (alongside age, sex, and height) predicted incident THR (Table 3). A well with an AUC of 0.83 (OR 5 per SD thickening, 95% CI 3.2-7.7). Adding baseline pain to the model increased AUC slightly to 0.85. 3D osteophyte load accounted for 25% of the variance in cortical thickness within the ROI (CTh in ROI = 1.2-0.01*osteophyte load, *p* < .0001, adj R^2^ = 0.25). While osteophytes did not fully explain cortical thickening, THR prediction in ROC analysis was the same whether 3D osteophyte load or CTh was modeled (AUC 0.83). Thinning of the joint space was only weakly associated with cortical thickening in cases (not controls) explaining 6% of the variance in CTh (CTh in ROI = 1.4-0.05*mJSW, *p* = .03, adj R^2^ = 0.06).

Patients heading for THR had specific focal and highly-conserved 3D structural changes of femoral head thickening by up to 70% at superior contact surface/articular margin, closely matching changes that we found previously in patients with OA from the UK^18^ (Figure 2).

### Self-reported hip pain performance

The percentage of the entire AGES-Reykjavik cohort (*n* = 3133) reporting hip pain at baseline was 24.3%. Similarly, the percentage reporting hip pain at baseline from our 258-participant sample was 26.0%. Only 45% (33/74) of patients destined for THR reported hip pain at baseline. The presence of hip pain was a poor-to-marginal predictor of THR (cross-validated AUC = 0.63). Hip pain alone had poor sensitivity for THR of 44.6% (33.8-55.9), in contrast to any imaging measure (Table 4A). The presence of self-reported baseline hip pain demonstrated very poor rule-generally in accuracy (UI+ 0.28) and good rule-out accuracy for THR (UI− 0.72).^9^

### Imaging performance

All imaging investigations ranged in clinical UI from good to excellent (Table 4B), except rule-out accuracy for mJSW which was adequate. Adding any radiological measurement to hip pain alone greatly enhanced the prediction of THR (Table 4B). Adding a simple distance measure (2D mJSW) improved AUC discrimination from 0.63 to 0.78 (*p* = .02). K&L grade alone gave an AUC of 0.85 (OR 6.30 per incremental K&L grade, 95% CI 4.0*-*10.0), unchanged with or without inclusion of baseline pain. K&L gave better discrimination than mJSW alone (0.85 vs 0.77 *p* = .0056). 2D K&L grading and 3D CTh seem to provide different information, as manifested by excellent (and optimal) prediction of THR in a model containing; pain, K&L grade and 3D CTh (AUC = 0.88, Table 3).

**Table 3:** Predicting total hip replacement for osteoarthritis within 8 yr; using baseline hip pain alone vs adding imaging tests. Comparisons of AUC of the various models), model *n* = 258.

**Model (*n* = 258)** **predicting THR**	**AUC (95% CI)**	**Cross-** **validated AUC**	**OR per SD (95% CI)**	**Difference between using imaging vs hip pain only (*χ*** ^**2**^ **)**	**Imaging vs hip pain only prob >*χ*** ^**2**^
**Hip pain only**	0.70 (0.62-0.78)	0.63	2.21 (1.68-2.91)	–	–
**mJSW only**	0.79 (0.72-0.86)	0.77	0.26 (0.17-0.39)	–	–
**^i^Hip pain + mJSW**	0.80 (0.73-0.87)	0.78	–	5.75	0.0164
**K&L grade only**	0.87 (0.81-0.92)	0.85	6.30 (3.96-9.99)	–	–
**^ii^Hip pain + K&L**	0.87 (0.82-0.93)	0.85	–	20.35	0.0000
**Cortical thickness in femoral patch (** **Figure 1** **)**	0.83 (0.77-0.89)	0.81	5.00 (3.24-7.71)	–	–
**^iii^Hip pain + CTh**	0.85 (0.79-0.91)	0.83	–	14.30	0.0002
**hip pain + K&L + CTh**	0.90 (0.85-0.95)	0.88	–	26.80	0.0000

i vs ii, χ^2^ = 9.25, *p* = .0024; i vs iii, χ^2^ = 2.21, *p* = .1375; ii vs iii, χ^2^ = 0.48, *p* = .4881.

Abbreviations: AUC, area under the curve; CTh, cortical thickness; K&L, Kellgren & Lawrence score; mJSW, minimum joint space width; OR, odds ratio per standard deviation (SD); ROC, receiver operating characteristic; χ^2^, chi-square test.

## Discussion

Primary care health professionals such as general practitioners managing patients with hip OA have a central role in referring individuals who might benefit from the *operation of the century*, as THR has been billed.^25^ It is appreciated that the decision to refer for this operation could be aided by appropriate risk stratification tests with positive clinical utility.^9^ Any test with negative clinical utility would also help health professionals reassure patients with hip OA who are *not* likely to require THR assessment in the near term, which has utility when choosing non-surgical options for hip OA. A decade after the landmark paper by Kim et al., it is surprising that the NICE OA guideline committee—perhaps influenced by cohort findings of discordance between hip pain and radiographic hip OA—elected to remove routine radiography from OA care pathways.^13^ Our findings do not support the guidance’s characterization of radiographs as “*unnecessary.*” Future guideline revisions might benefit from revisiting the final paragraph of the BMJ editorial that accompanied the paper, which prudently delineated the role of imaging in the management of patients with hip pain.^26^

When it comes to the role of imaging in assessing patients with hip pain, anteroposterior hip radiographs commonly demonstrate structural features of OA—reduced joint space width, osteophytes, and femoral head subchondral sclerosis.^10^^,^^11^^,^^27^^,^^28^ Opinion differs about routine imaging: some guidance advises against routine radiographs for suspected OA^16^ based on^13^ while others continue to recommend radiographs to help differentiate among the common causes of adult hip pain.^29^ In the present nested case-control study of older adults from Iceland, we quantified clinically and surgically relevant features of hip OA using pelvic CT, focusing on: (1) 2D K&L grading, (2) 2D minimum joint space width (mJSW), and (3) 3D CTh of the proximal femur. In participants in their mid-70s who reported hip pain at baseline, these imaging measures had useful prognostic performance for predicting THR within roughly three years. In our sample, a report indicating K&L ≥2 in a painful hip correctly identified most people who went on to THR and correctly reassured most who did not (Table 4B). These findings align with larger UK Biobank analyses which identified that osteophyte burden and OA grades clearly associated with prevalent hip pain and with later clinical outcomes including hospital-diagnosed OA and THR.^30^ Notably, the UK analyses suggested that 2D osteophyte area related more strongly to pain than mJSW alone, implying that different radiographic features contribute variably to symptoms.^30^^,^^31^

**Table 4:** (A) Performance of hip pain alone in predicting THR. (B) Effects of adding any imaging test result to patients presenting with hip pain on clinical utility index (7)a, *n* = 52 (33 from case group and 19 from control group).

**A. Baseline pain status predicting THR (*n* = 258)**	**Sensitivity**	**Specificity**	**PPV**	**NPV**	**PLR**	**NLR**
**Hip pain (WOMAC)** **UI+ 0.28/UI− 0.72**	44.6% (33.8-55.9)	89.7% (84.4-93.3)	63.5% (49.9-75.2)	80.1% (74.1-85.0)	4.3 (2.6-7.1)	0.60 (0.5-0.76)
**B. Utility of imaging for those presenting with pain (*n* = 52)**						
**+K&L grade** ^**a**^ **UI+ 0.82/UI− 0.74**	84.8% (69.1-93.3)	94.7% (75.4-99.1)	96.6% (82.8-99.4)	78.3% (58.1-90.3)	16.1 (2.4-109.2)	0.2 (0.1-0.4)
**+2D mJSW** ^**b**^ **UI+ 0.70/UI− 0.61**	75.8% (59.0-87.2)	89.5% (68.6-97.1)	92.6% (76.6-97.9)	68.0% (48.4-82.8)	7.2 (1.9-27.1)	0.27 (0.1-0.5)
**+CT-derived 3D CTh** ^**c**^ **UI+ 0.79 / UI− 0.71** ^**a**^	81.8% (65.6-91.4)	94.7% (75.4-99.1)	96.4% (82.3-99.4)	75.0% (55.1-88.8)	15.5 (2.3-105.5)	0.2 (0.1-0.4)

a Outcome by a K&L grade of ≥2.

b For females JSW <1.24 mm and males <1.73 mm.

c For females, femoral cortical thickness >1.2 mm and males >1.29 mm.

These diagnostic cut-offs were derived using JMP add-in (“ROC and pAUC Analysis” by D Meintrup). Qualitative interpretation of utility index (UI+ in prediction of positive outcome, UI− in prediction of negative outcome) from Mitchell, A (7) ≥0.81 excellent; ≥0.64 good; ≥0.49 adequate; ≥0.36 poor, and ≤0.36 very poor.

Abbreviations: K&L, Kellgren & Lawrence score; NLR, negative likelihood ratio; NPV, negative predictive value; PLR, positive likelihood ratio; PPV, positive predictive value.

Guidelines differ in emphasis. NICE Quality Standard QS87 (based on NG226) recommends against routine imaging to diagnose OA, stating there is limited evidence that imaging improves diagnostic management*.*^16^^,^^32^ We do not contend that every patient with hip pain requires imaging, but our data supports many others in confirming that imaging can materially inform management when the test result would change care^26^—for example, when assessing a patient with hip pain for suspected “severe OA” (a critical part of patient and clinician decision making^33^), considering referral for surgery,^33^ excluding alternative pathology, or planning interventions.^26^ In these scenarios, an imaging report showing definite radiographic OA in the painful hip (eg, K&L ≥ 2) had good confirmatory and screening utility for subsequent THR (Table 4B). Based on our data, with imaging reports available, the health professional seeing the individual with hip pain could correctly refer 85% (28/33) of people who would go on to require THR within 3 yr and correctly reassure 95% (18/19) that THR would not be performed. Knowing that these patients’ K&L score was ≥2 was excellent for confirmatory case finding (positive clinical UI 0.82 (0.70, 0.94)), and good for screening; that is, ruling out which patients would not require THR (negative clinical UI 0.74 (0.62, 0.87)). Simpler measures (performed manually in seconds) had similar clinical utility: a minimum joint space ≤1.24 mm for women and ≤1.73 mm for men would correctly identify 76% (25/33) and reassure 89% (17/19 Table 4B).

The multi-color patch in the hip model in Figure 2 is a critical anatomical zone when considering where the bone surfaces of hip and socket meet. Our 3D mapping technique has therefore uncovered a new marker of surgery-destined severe OA. Bone thickness (CTh) values ≥1.20 mm for women and ≥1.29 mm for men in the patch would correctly identify 82% (27/33) of people with painful hips destined for THR within roughly 3 yr and reassure 95% (18/19 Table 4B) that they would not end up having surgery.^33^ It is not yet known if this focal thickening relates to joint shape. Morphological determinants (sex, size, and shape mode) of 3D femoral CTh in older people have previously been identified.^34^^,^^35^ In further work, it would be interesting to explore relationships between femoroacetabular impingement (FAI)-relevant shapes (such as cam deformity) and the focally thickened areas identified in patients with surgically relevant OA.

We retained the full K&L 0-4 scale because it preserved prognostic discrimination across radiographic severity. Dichotomising K&L grade (eg, into <2 vs ≥2) is useful for simple rule-in reporting—the general practitioner requester receiving a report that a painful hip has moderate-severe OA (K&L ≥ 2) can then readily confirm causality and escalate management.^30^ However, this binary approach sacrifices information: the risk of future THR increases markedly with each K&L increment (near-exponentially in our data), so models using the full 0-4 scale provide substantially better discrimination for individual risk stratification. Thus, while a dichotomous K&L ≥2 flag is practical for immediate clinical decisions, maintaining the 0-4 grading on the report better informs prognosis and allows more nuanced stratification (eg, prioritizing K&L 3-4 for expedited referral as per German National guidelines). Unlike NICE QS87 (but like the UK Royal College of Surgeons, 2017) the German 2021 guidelines consider radiographs to be critical, “THR should be performed solely with radiologically demonstrated advanced OA of the hip (K&L grade 3-4), after at least 3 mo of conservative treatment, and in the presence of high subjective distress due to symptoms arising from the affected hip joint.” The rule-out performance of hip imaging is also clinically relevant: Logishetty et al. (2025) reported that patients undergoing total hip arthroplasty with minimal or no radiographic OA had lower postoperative function than typical THA patients and recommended both low-dose CT imaging and diagnostic injection to aid decision-making.^36^ Since THR has demonstrated superiority over conservative treatment for “severe hip OA” in a recent large RCT, imaging that helps define “severe” disease may meaningfully inform referral and treatment prioritization.^37^

Formal K&L grading is rarely done in clinical practice and is subject to intra- and inter-observer variability. Relying on a simpler descriptive OA grading may not be any better; one of us audited hospital radiology reports and found only 53% reliability from routine clinical hip radiograph reports in allowing primary care doctors to determine which patients should be referred for orthopedic surgery assessment (4% with no comment at all, 10% of reports that could have led to missed referral, and 33% of reports that could have led to inappropriate referral, TDT *personal communication*). While semi-automated 3D bone mapping from CT has no prerequisite for radiological expertise, the higher radiation dose, availability, processing steps and cost of CT will clearly favor 2D radiographs. Since formal K&L grading requires specific expertise, we also tested the FDA-approved clinical trial gold standard of minimum superior joint space width measure which takes moments using any modern PACS reporting station.^38^ This showed mJSW to be highly predictive of THR in symptomatic individuals, although not quite as good as K&L grade (AUC 0.77 compared to 0.85, Table 3).

An important caveat is that these joint space measurements are only correct for supine radiographs (DRRs) digitally reconstructed from CT and might be different for plain radiographs; we lacked images from both techniques to allow comparisons.^39^ Supine acquisition might yield slightly larger absolute JSW values than weight-bearing imaging^17^^,^^21^^,^^40^ associations with THR are unlikely to be driven by supine acquisition alone. We note this as a limitation and recommend external validation against weight-bearing measures. As a clinical strategy this measure may also be limited by inter-subject variability since normal hip mJSW does vary across a 5 mm range.^41^ Since hip OA imaging is of most utility as a stratification test to aid with referral vs non-referral, a future priority is to extend our analysis to that larger AGES-Reykjavik sample. These aims are in keeping with those of the Quantitative Imaging Biomarker Alliance https://www.rsna.org/QIBA/. It would also be interesting to study the relationships between pain and imaging in the 3113 randomly sampled healthy Icelandic volunteers to compare with Kim et al.’s younger US volunteers selected for their knee OA risk.^13^

There are other limitations to our study. Receiver operating characteristic analysis in matched case-control studies may necessitate adjustment for potentially confounding covariates, but we used standard unadjusted research methods. We lack information from clinical examination of the hip in the participants and a lack of data on patient surgical/conservative treatment preference. Finally, Iceland has a very high prevalence (5-fold higher than in southern Scandinavia) of radiological primary hip OA.^28^ Hence, generalizability beyond Icelandic population is unknown, although this study was the validation of an original discovery made in a UK cohort so it is likely that the findings are robust at least to the UK and Iceland.^18^ Since the cohort was racially homogenous, comprised of white Icelandic individuals, this inevitably limits applicability to other groups.^38^

Previously, we demonstrated that the inclusion of detailed 3D joint space parameters alongside K&L grade delivered an 18% improvement on mJSW alone (AUC of 0.86 compared to 0.73, respectively) in a predictive model for the future THR in the same study cohort.^19^ In the present study, we are now able to propose that the 3D appearance of femoral cortical thickening can help characterize OA destined for surgery. Faber et al.^31^ found important associations between osteophyte size or location and hip pain.^31^ In our cases, destined for THR, osteophytes accounted for 25% of the variance in CTh within the 3D ROI (CTh in ROI = 1.2-0.01*Osteophyte load, *p* < .0001, adj R^2^ = 0.25, Figure 2). While osteophytes did not fully explain cortical thickening, THR prediction in ROC analysis was the same whether 3D osteophyte load or CTh was modeled (AUC 0.83). We suspect that painful osteophyte-derived cortical thickening at the femoral head (blue in Figure 2) is a key pathological entity in painful hip OA. The 3D bone thickening we observed in Figure 2 was particularly prominent at the femoral contact area with the acetabular labrum, where osteophytes typically form and in the crescent of bone that is preferentially loaded during walking and sitting.^42^ If such features are present in younger patients, these 3D imaging techniques might provide an opportunity to identify those whose nascent OA is amenable to emerging, for example, stem cell therapies.^43^ Irrespective of the underlying cause of OA, this characteristic region of femoral cortical thickening not only predicted THR with an OR of 5.0 or each SD thicker (95% CI 3.2*-*7.7, Table 3), but was also predictive of baseline hip pain with OR 2.1 (95% CI 1.5*-*3.1, Table 5).

**Table 5:** Odds ratios of radiographic osteoarthritis for prevalent hip pain.

**K&L classification**	**Number of hips**	**K&L grade**	**OR (95% CI)**	***p* value**
**Doubtful**	66	1	0.83^*^ (0.24-2.28)	.7363
**Mild**	45	2	1.98^*^ (0.69-4.95)	.1914
**Moderate/severe**	50	3 + 4	12.22^*^ (6.00-25.46)	<.0001
Moderate^***^	40	3	8.92 (4.10-19.42)	<.0001
Severe^***^	10	4	50.78 (10.05-256.58)	<.0001
**mJSW**	458	–	2.68^**^ (1.64-4.36)	<.0001
**CTh**	258	–	2.14^**^ (1.46-3.12)	<.0001

In addition, multiple linear regression was calculated to predict mJSW based on age, sex, and hip pain, replicating Dougaros et al. 1996 (29). A significant regression equation was found (F(3,454) = 26.1 *p* < .0001), with an R^2^ of 0.15. Participants predicted mJSW was equal to 3.12 mm − 0.35 (pain) − 0.02 (age) + 0.22 (sex), where pain was coded as 1 = yes, 2 = no, age was measured in years and sex was coded as 1 = male, female = 2. mJSW was 0.35 mm thinner in those with hip pain and males had 0.22 mm wider mJSW than females.

Abbreviations: CTh, cortical thickness; K&L, Kellgren & Lawrence score; mJSW, minimum joint space width; OR, odds ratio per standard deviation (SD). ^*^versus none ^**^per SD narrowing (mJSW) or thickening (CTh) ^***^individual K&L grades.

### Conclusion

In conclusion, these data support targeted imaging assessment for OA in older individuals presenting with hip pain when the result would influence management, since imaging features (including K&L grade, mJSW thresholds, and quantitative osteophyte or 3D cortical measures) improve prediction of surgically-relevant disease and can aid referral, reassurance, and treatment decisions.

## Data Availability

The data underlying this article will be shared on reasonable request to the corresponding author.
